# IKBKE-driven TPL2 and MEK1 phosphorylations sustain constitutive ERK1/2 activation in tumor cells

**DOI:** 10.17179/excli2021-4578

**Published:** 2022-02-18

**Authors:** Serkan Ismail Göktuna

**Affiliations:** 1 Department of Molecular Biology and Genetics, Bilkent University, Ankara, Turkey; 2 National Nanotechnology Research Center (UNAM), Bilkent University, Ankara, Turkey; 3 Laboratory of Medical Chemistry, Interdisciplinary Genomics and Genoproteomics Research Center (GIGA), University of Liege, Liege, Belgium

**Keywords:** cancer cells, signal transduction, IKKepsilon (IKBKE), TPL2 (MAP3K8), MEK1 (MAP2K1), ERK1/2 (MAPK1/2)

## Abstract

IKBKE have been associated with numerous cancers. As a result, IKBKE have emerged as potential target for cancer therapy. Accumulating evidence support that IKBKE orchestrate tumor cell survival in cancers. Here we evaluated the possible link between IKBKE and ERK phosphorylation. The effects of IKBKE silencing on MAPK activation in tumor vs. normal cells were evaluated via WB and RT-PCR. Ectopically expressed IKBKE, TPL2 or MEK1 constructs were used to examine the possible interactions among them via co-IP. *In vitro* kinase assays were performed to understand nature of the observed interactions. In tumors, IKBKE regulates MEK/ERK constitutive activations *in vitro *and* in vivo*. IKBKE and TPL2 physically interact and this interaction leads to TPL2 phosphorylation. We describe here a novel regulatory link between IKBKE and constitutive ERK1/2 activation in tumor cells. This new circuitry may be relevant for tumor cell survival in various malignancies.

## Introduction

Mitogen activated protein kinases (MAPKs) are involved in many vital processes related to cell growth and survival. MAPKs like ERK1/2 are activated by growth factors and other stimuli, directing these signals to effector proteins through a series of signaling cascades (Lemmon and Schlessinger, 2010[33]). Most of the signal transduction machinery is induced by growth hormone ligand binding to RTKs, which channel the information to some relay elements like Ras or similar activators of different MAPK signaling schemes. Subsequently, MAP3Ks like Raf1 can phosphorylate MEK1 (a MAP2K) which further activates ERK1/2 (a MAPK) through a series of sequential phosphorylation events (Terrell and Morrison, 2019[51]). While MEK1 and MEK2 are only known kinases for ERK1/2 activation (Dang et al., 1998[7]), MEK1 can be activated by a RAF family of MAP3 kinases (such as Braf, Raf1 (Craf), Araf) in various tissues and physiological conditions (Huang et al., 1993[25]; Matallanas et al., 2011[39]). Other studies revealed that MEK1 can be activated by alternative kinases such as TPL2 (also referred to MAP3K8) as in blood cells, Mos in oocytes and MEKK1 in macrophages (Salmeron et al., 1996[44]; Verlhac et al., 2000[54]; Xu et al., 1995[60]). Once activated by MEK1, ERK1/2 can phosphorylate a number of downstream effector proteins (like c-Jun, c-Myc, MSK1, RSK1, MCL1 and others) many of which act as transcription factors orchestrating key processes in proliferation, inflammation and cell survival (Lavoie et al., 2020[28]).

ERK1/2 phospho-activation was observed in various carcinomas where it is crucial in cancer cell survival and proliferation. Resulting from various mutations and alterations in growth factor receptor signaling pathways, constitutive ERK1/2/MAPK activation is a common theme in many tumor cells (Dhillon et al., 2007[9]). While the activation of RTK-induced signaling pathways may require the presence of growth factors or intact ECM attachment in normal cells, many of these pathways are deregulated in cancers so that downstream signaling may progress independently of these factors (Lemmon and Schlessinger, 2010[33]). Various alterations in RTK signaling result in constitutive MAPK activation. For instance, RTK gene duplications, point mutations in receptor/effector genes enhancing ligand-independent coupling, Ras-activating mutations (Kras^G12D^ in colorectal cancer), Raf activating mutations (Braf^V600E^ in melanomas) are some examples of the most commonly observed dysregulated signaling pathways resulting in constitutive and signal-independent MAPK activation (Friday and Adjei. 2008[13]). Ras/Raf/MEK/ERK1/2 pathway is known to be the predominant path for MAPK activation in growth factor-induced signal transduction schemes in various tissues. However, in certain tissues or in specific cellular processes, other MAP3Ks may replace RAFs for MEK1 activation. For instance, TPL2 is also known to activate MEK1 in various physiological conditions and in various cancers (Lee et al., 2015[30]). More specifically, LPS or IL-17-driven immune cells activation predominantly depends on TPL2-mediated MEK/ERK phosphorylations (Kim et al., 2013[26]; Xu et al., 2018[59]).

TPL2 activity is associated with the regulation of inflammatory pathways in immune cells and in cells responding to inflammatory cytokines (Lee et al., 2017[31]; Virla et al., 2018[55]). Especially, the involvement of TPL2, its upstream activators like AKT or IKKβ (IKBKB) and phospho-regulatory sites on TPL2 like T290 and S400 are fully described in LPS-induced MEK/ERK1/2 activation (Beinke et al., 2004[1]; Cho and Tsichlis, 2005[6]; Gantke et al., 2011[14]; Robinson et al., 2007[42]; Roget et al., 2012[43]). Priming phosphorylation by these kinases as well as autophosphorylation of other residues may be required for full TPL2 activation. Although the importance of some of these phosphorylation events are well described in the literature, other proteins may regulate TPL2 activation under various circumstances (Virla et al., 2018[55]). Likewise, *Helicobacter hepaticus* challenge on macrophages or pathogen-associated molecular patterns (PAMPs) challenge on airway epithelium were also shown to activate ERK1/2 through TPL2 (Martel et al., 2013[38]; Tomczak et al., 2006[53]). Besides, TPL2 may be relevant in a positive feedback loop in inflammatory responses through p38/MAPK activation (Pattison et al., 2016[41]). Similarly, TPL2 is also involved in JNK/MAPK activation in B lymphocytes (Voigt et al., 2020[56]). All these studies collectively suggested that TPL2 is involved in the regulation of diverse MAPK signaling schemes in inflammatory cells.

IKK-related kinase IKBKE (IKKɛ) has been shown to be involved in the pathogenesis of various cancers (Durand et al., 2018[11]; Yin et al., 2020[61]). In this context, we described some novel roles for IKKɛ in cancer survival and in mTOR/S6K activation in colorectal cancer (Göktuna et al., 2016[19]; Göktuna, 2018[17]). In these studies, we observed that constitutive AKT and ERK phosphorylations were seriously diminished in tumor cells lacking IKKɛ (Göktuna et al., 2016[19]). Therefore, our and other works have suggested that constitutive levels of AKT phosphorylation is directly regulated by IKKɛ in colorectal tumors (Xie et al., 2011[58]). Nevertheless, the nature of IKKɛ-dependent ERK1/2 activation was not addressed in these studies. To clarify whether or not the IKKɛ-dependent ERK activation is specific to colorectal cancer cells, we aimed to further explore the nature of IKKɛ-dependent signaling events leading to constitutive ERK1/2 activation in various tumor cells.

## Materials and Methods

### Experimental procedures

#### Mice, cell lines and tissue culture

IEC lysates obtained from previous studies (Göktuna et al., 2016[19]) were also used in this study. Villin-cre-ER^T2^-Ctnnb1^+/lox(ex3)^ (*β-cat^c.a.^*) mice were kindly gifted by Prof. Florian Greten (GSH, Frankfurt, Germany); C57BL/6J-Apc^Min^/J (*Apc^+/min^*) and B6.Cg-Ikbke^tm1Tman^/J (*Ikbke^-/-^*) mice were purchased from The Jackson Laboratory (USA). All experimental procedures from the previous study were approved by University of Liege Animal Ethics Committee and no additional animals were used here. 

Transformed MEF cells (MEF-T) (kindly provided by Dr. Emmanuel Dejardin), HEK293, RAW264.7 and HeLa (ATCC, USA) cells were maintained in DMEM medium (Lonza, Switzerland); BMDMs were maintained in RPMI1640 medium (Lonza, Switzerland) supplemented with L929 medium (Göktuna et al. 2014[18]); MCF-7 (ATCC, USA) cells were cultured in EMEM (without phenol red) medium (Lonza, Switzerland); SW480 cells (ATCC, USA) were cultured in L-15 medium (Sigma, USA); DLD-1 and HCT116 (ATCC, USA) cells were maintained in McCoy 5A medium (Lonza, Switzerland). All these media were supplemented with 10 % FCS (Sigma, USA), 1 % L-glutamine (Lonza, Switzerland) and 1 % penicillin-streptomycin (Lonza, Switzerland).

Unless otherwise stated, in all cellular stimulation experiments LPS was used in 10 ng/ml; Poly I:C was used in 10 µg/ml and TNFα was used in 20 ng/ml concentration at indicated time points. For stimulation of mice intestinal epithelia, a sublethal dose of 0.1 mg/kg LPS (Guma et al., 2011[21]) was injected intraperitoneally to the mice and small intestines were collected at indicated time points (0-8h) after injections.

Intestinal epithelial cells were collected from mice, as previously described (Göktuna et al., 2016[19]). Briefly, small intestines were collected after euthanizing mice. Intestines were flushed with PBS, then they were cut into three pieces (duodenum, jejunum and ileum) along their latitudinal axis. Duodenum was next minced into small pieces and incubated 10 minutes in HBSS (Lonza, Switzerland) supplemented with 30 mM EDTA at 37 °C with gentle agitation. Finally, epithelial cells were detached from lamina propria by vigorous vortexing at 1500 rpm for 30 seconds. Epithelial cell supernatants were quickly collected in another test tube and washed twice in ice cold PBS and pelleted in microcentrifuge tubes at 5000 rpm for 5 minutes. Finally, aspirated cell pellets were snap-frozen in liquid nitrogen and stored at -80 °C for later use.

#### Plasmids and transfections 

pcDNA3.1-HA-TPL2 and pcDNA3.1-GST-TPL2^367-467^ expression plasmids were generated from parent plasmids obtained from Addgene (USA). pCMV or pcDNA3 based Myc-IKKɛ^WT^ and Myc-IKKɛ^KD^ (K38A) expression plasmids were previously generated by our group (Chariot et al., 2002[5]).

siRNAs, bought from Eurogentec (Belgium), were used for human IKBKE (IKKɛ) or human MAP3K8 (TPL2) gene silencing. All transfections were carried out in Opti-MEM medium (Gibco/Thermo, Germany). Plasmid DNAs were transfected into mammalian cell lines by the use of Transfectin (Bio-Rad, USA) or TransIT-X2 (MirusBio, USA) transfection reagents while siRNA constructs were delivered into mammalian cells via Hiperfect (Qiagen, USA). All transfection protocols were carried according to procedures described by manufacturers. Gene silencing or ectopic expressions were further controlled via specific antibodies used in Western Blots (WB).

#### Western blotting, immuno-precipitations, Ras activation and kinase assays

All experimental procedures were performed as previously described (Göktuna et al., 2016[19]; Göktuna, 2018[17]). Shorty, crude cell lysates from various procedures were run on 8-12 % SDS-PAGE. These gels were then blotted on to PVDF membranes (Millipore, USA) and target proteins (coupled with specific primary and HRP-linked secondary antibodies) were visualized as specific bands by the use of chemiluminescence reagent (Pierce/Thermo, Germany) on X-ray film (Fujifilm, Japan). Immunoprecipitation (IP) reactions were performed with Protein A agarose beads (SCBT, USA) and specific antibodies. Briefly, cell lysates were incubated with agarose beads and antibody mixture by gentle rotation at 4° C for 4 hours. After 3 to 4 times washed with cell lysis buffer, these beads were used for WB or *in vitro* kinase assay. For *in vitro* kinase assay, endogenous or overexpressed kinases were pulled out from cell lysate by IP and incubated with the specific substrate protein (overexpressed or recombinant), cold ATP and radioactive ATP (^32^P-γ-ATP; Perkin Elmer, USA) for 30 minutes at 30 °C. Beads were boiled at 95 °C in SDS-PAGE loading buffer to stop the reaction and to extract proteins. Phosphorylated samples blotted on PVDF membranes via WB were then visualized via auto-radiography to detect relative kinase activity in each sample. For Ras activation assay, a Ras Activation Assay Biochem Kit was purchased from Cytoskeleton Inc. (Denver, USA) and all experiments were performed according to the manufacturer's protocol as previously described (Shostak et al., 2014[48]). Briefly, IECs harvested from β*-cat^c.a.^* mice were lysed with supplied lysis buffer and 300 μg total proteins were used for immunoprecipitation of active Ras protein (GTP-Ras) by Raf-RBD beads for 1 hour at 4 °C. GTP-Ras bound beads were washed twice with supplied washing buffer and added with 20 μl Laemmli buffer before boiling samples in preparation to WB. Blotted membranes were used for immunoblotting with Ras specific antibody and visualized by ECL.

Antibodies used: anti-IKKɛ #I4907 (Sigma), anti-TBK1 #3013 (CST), anti-COT/TPL2 (SCBT), anti-ERK1/2 (SCBT), anti-p-ERK1/2 (CST), anti-MEK1 (SCBT), anti-p-MEK1 (CST), anti-α-tubulin #T9026 (Sigma), anti-IKKβ #05-535 (Upstate/Millipore), anti-Myc #sc-764 (SCBT), anti-HA #H3663 (Sigma) and anti-pan-RAS (Cytoskeleton).

#### Gene expression analysis via RNA-Seq and RT-PCR

Total RNAs were extracted using the E.Z.N.A Total RNA kit (Omega Biotek, USA). cDNAs were synthesized by the use of Revert aid H minus reverse transcriptase kit (Thermo, Germany). RT-PCR analysis was performed by using SYBR Premix Ex Taq (Takara, Japan) on a LightCycler 480 Real-Time PCR System (Roche, Germany). Primer sequences used in this study are human-MAP3K8-F 5'-ATGGAGTACATGAGCACTGGA-3'; human-MAP3K8-R 5'-GCTGGCTCTTCACTTGCATAAAG. Gene expression profiling of tumor tissue was performed using an Illumina RNA-Seq array as previously described (Göktuna et al., 2016[19]). Raw data was processed and expression data was normalized by using TopHat and Cufflinks software packages in R.

For bioinformatic analyses related to phosphorylation sites, predictions were carried by the help of Phosphosite Plus website (Hornbeck et al., 2015[23]) prediction algorithm and Phosphonet Kinase Predictor (Kinexus Bioinformatics) algorithms (available at http://www.phosphonet.ca). For TCGA patient data, online webtools available from GEPIA were used (Tang et al., 2017[50]).

#### Statistics

For statistical analysis of the expression data, Student's t-test was used and p values less than 0.05 was considered significant. All statistical analyses were performed by using Prism 5 Software (Graphpad, USA).

## Results

### Constitutive ERK1/2 activations of tumor cells are abrogated in the absence of IKBKE

**Figure 1:**
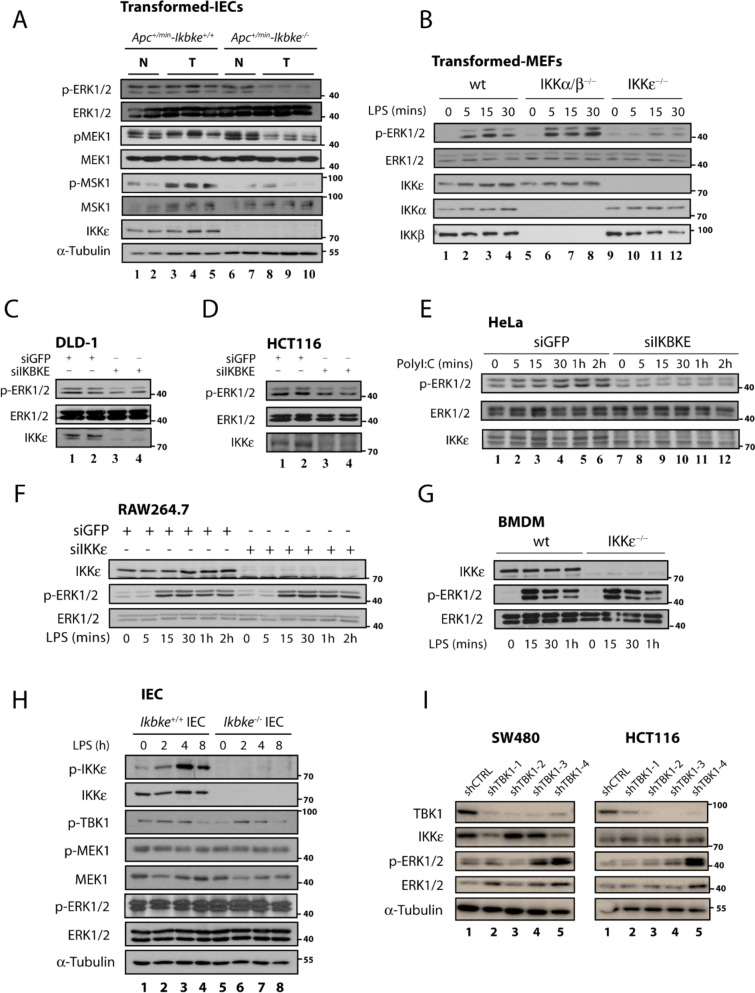
ERK1/2 activations are abrogated in IKKɛ deficient tumor cells from various tissues. (A) ERK1/2 and MEK1 constitutive phosphorylations are diminished in IKBKE deficient IECs from* Apc^+/min^* mice but only in tumor samples. (B) Transformed MEF cells deficient of only IKBKE (but not in IKKα or IKKβ) also show abrogated ERK1/2 phosphorylation (where different genotypes of MEF cells were stimulated 0-30 minutes, at indicated time points, with 10 ng/ml LPS). (C-E) Tumor cell lines from various epithelial neoplasia also show diminished ERK1/2 phosphorylation upon loss of IKBKE via siRNA knockdown after stimulated with respective ligands (where DLD-1 and HCT116 cells were stimulated 0-30 minutes, at indicated time points, with 10 ng/ml LPS and HeLa cells were treated 0-2 hours, at indicated time points, with 10 µg/ml Poly I:C). (F-H) On the contrary, ERK1/2 activations in normal cells were not affected from IKKɛ deficiency. (F) RAW264.7 cells and (G) BMDMs were treated with 10 ng/ml LPS, as indicated in each panel while (H) *Ikbke* wt or ko mice were injected with 0.1 mg/kg LPS 0-8 h, at indicated time points, for intestinal epithelial cell stimulation. ERK1/2 phosphorylation was measured by WB using a phospho-specific antibody. (I) Impact of TBK1 depletion on ERK1/2 phosphorylations were evaluated in CRC cell lines. To do this, ERK1/2 phosphorylation was examined in lentiviral shRNA (shCTRL vs shTBK1 (1-4)) transfected SW480 and HCT116 cells via WB.

We have previously reported that IKBKE deficiency specifically diminishes constitutive AKT and ERK1/2 activations in Wnt-transformed epithelial cells which succumbs to increased apoptotic rates in the absence of these survival pathways (Göktuna et al., 2016[19]). Here we aimed to better understand the link between IKKɛ and ERK1/2 activation by assessing the activation of both kinases in various normal and tumor cells treated or not with appropriate ligands (LPS and Poly I:C) (Figure 1A-E(Fig. 1)). First, we have re-examined IECs samples from *Apc^+/min^-Ikbke^wt^* vs. *Apc^+/min^-Ikbke^-/-^* mice we generated in our previous study (Göktuna et al., 2016[19]) to examine ERK1/2 phosphorylation via WB (Figure 1A(Fig. 1)). Either* Apc* loss or β-catenin stabilization models similarly activated Wnt-driven transformation in intestines. We have observed that basal MEK1, ERK1/2 and MSK1 (an ERK1/2 downstream target) phosphorylations were all diminished only in Wnt-transformed IECs in the absence of IKKɛ (Figure 1A(Fig. 1), lanes 8-10). Peculiarly, no such phenomenon was observed in IKKɛ deficient normal epithelia (Figure 1A(Fig. 1), lanes 6&7). Similar to *in vivo* samples, we found that IKBKE knockdown prominently diminishes constitutive ERK1/2 activation in other transformed/tumor cell lines as well (Figure 1B-F(Fig. 1)). LPS-stimulated (10 ng/ml) MEF-T, DLD-1 or HCT116 cells all showed a reduced ERK1/2 phosphorylation upon IKKɛ loss (Figure 1B-D(Fig. 1)). Additionally, these results were also relevant in cancer cells from other tissues, such as HeLa upon poly I:C (10 µg/ml) treatments (Figures 1E(Fig. 1)) and MCF-7 cells (since estradiol stimulation does not induce ERK1/2 activation, only basal ERK1/2 phosphorylation was affected; not shown). Almost in all transformed or cancer cell lines we examined, we observed a reduced ERK1/2 baseline or constitutive phosphorylations in the absence of IKBKE. Although some signal-dependent increase in ERK1/2 phosphorylations occurred in some cell lines in the absence of IKKɛ, these changes were still considerably lower than cells with IKKɛ. However, no such relations were observed in signal-dependent phosphorylation of ERK1/2 in normal cells, such as LPS stimulated mouse RAW267.4 cells or in intestinal epithelia (via i.p. injection) and BMDMs obtained from C57BL/6 mice (Figure 1F-G(Fig. 1)). Similarly, normal IECs obtained from LPS-stimulated mice with IKKɛ deficiency were not showing any difference in basal or signal activated ERK1/2 phosphorylation (Figure 1H(Fig. 1)). These results were confirming the absence of ERK1/2 phospho-regulation in normal IECs samples from *Apc+/min-Ikbke^-/-^* mice (Figure 1A(Fig. 1)). To evaluate any redundant function for TBK1 (a functionally and structurally similar IKK-related kinase) on ERK1/2 constitutive phosphorylation, we silenced TBK1 in SW480 or HCT116 cell lines via lentiviral shRNA constructs (Figure 1I(Fig. 1)). Unlike to IKKɛ, TBK1 deficiency in CRC cells did not impact baseline ERK1/2 phosphorylation in the same manner (as evident by higher ERK1/2 phosphorylation in some shTBK1 constructs). Therefore, these observations collectively pointed that IKKɛ curiously maintains the basal and constitutive levels of ERK1/2 activations only in tumor cell lines but has no effect on signal dependent activation of ERK1/2 in any cell types. 

### TPL2 is stabilized upon IKBKE loss 

**Figure 2:**
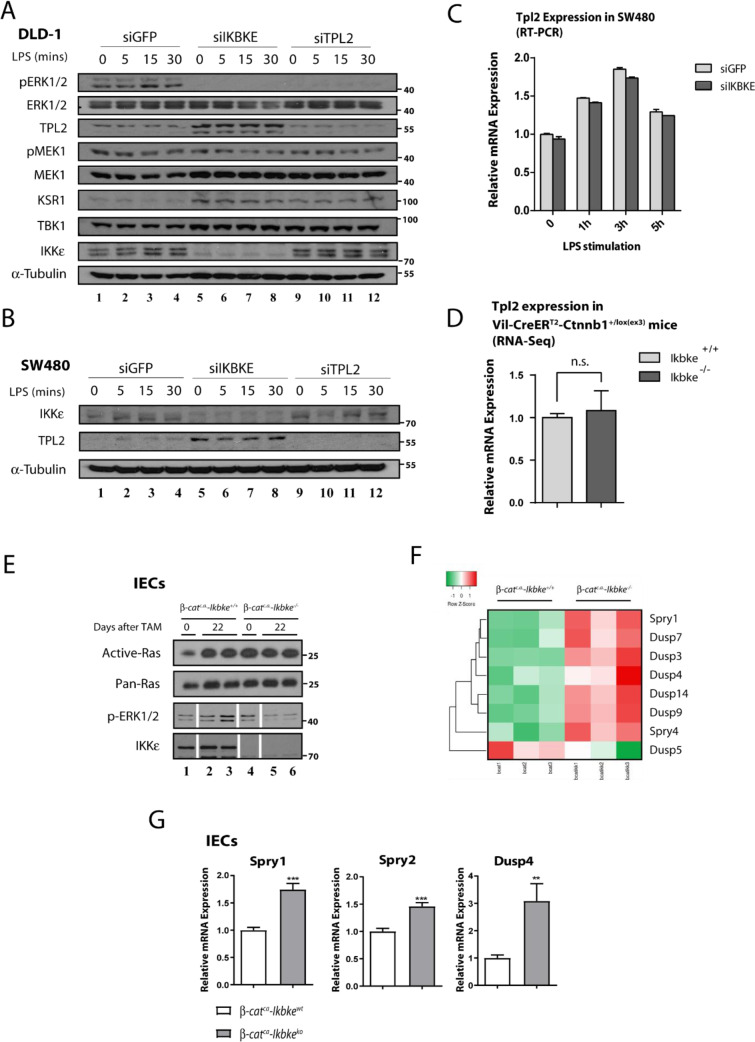
TPL2 protein levels are stabilized in CRC cell lines upon IKBKE loss. MEK1 and ERK1/2 phosphorylations are affected from loss of IKKɛ or TPL2 by siRNA knockdown in DLD-1 cells, as measured by WB using the indicated antibodies. Additionally, TPL2 protein stabilization upon loss of IKKɛ both in DLD-1 (A) and (B) SW480 cells (DLD-1 cells were stimulated 0-5 hours; SW480 cells were stimulated 0-30 minutes with 10 ng/ml LPS, at indicated time points). TPL2 RNA expression is not affected in (C) upon IKKɛ loss in SW480 cells or (D) in IKBKE-deficient Wnt-transformed colonic epithelia from *β-cat^c.a.^* mice. (E) Ras activity assay carried in samples from *β-cat^c.a.^* mice (0 or 22 days after tamoxifen induction) show that Ras activity is not affected by IKBKE deficiency (white bars in control loading show splicing points where unused samples in Ras-activity assay were excluded). Unedited WB bands are illustrated in Supplementary Figure 1). (F) Significantly and differentially regulated RTK/MAPK pathway phosphatase expressions in Wnt-transformed IECs were visualized via heatmap from the RNA-seq data. (G) Increased expression of indicated RTK/MAPK pathway phosphatases in IKBKE-deficient Wnt-transformed IECs were further confirmed with RT-PCR (**: p<0.01; ***: p<0.001 by Student's t-test).

To further investigate the nature of regulation on ERK1/2 upon IKKɛ loss in Wnt-transformed epithelial cells, we analyzed some ERK1/2 upstream kinases in IKKɛ deficient SW480 and DLD-1 cells. ERK1/2 is phosphorylated by MEK1 or MEK2 kinases for activation, yet MEK1 is phosphorylated by a number of different kinases including RAF-1, BRAF, ARAF, MKK1, Mos and TPL2 in different cells (Lavoie et al., 2020[28]). We concentrated our efforts on identifying downstream kinases mediating IKKɛ-dependent MEK/ERK1/2 activations. For this purpose, we checked the activity of two of the most common MAP3 kinases for MEK, namely RAF and TPL2, upon IKKɛ loss. First, we addressed the effect of TPL2 loss on ERK1/2 activation in CRC cell lines subjected to siRNA silencing (against IKKɛ or TPL2) and LPS stimulation (Figure 2A-B(Fig. 2)). We observed diminished ERK1/2 phosphorylation in DLD-1 cells upon IKKɛ or TPL2 deficiency (Figure 2A(Fig. 2), lanes 5-12). Moreover, MEK1 phosphorylations were also reduced upon IKKɛ or TPL2 deficiency in DLD-1 yet phospho-MEK1 did not totally disappear (Figure 2A(Fig. 2)). More interestingly, TPL2 protein levels were stabilized upon IKKɛ loss in DLD-1 and SW40 cells (Figure 2A&B(Fig. 2), lanes 5-8). Then, we also checked the expression of a scaffold/adapter protein KSR1 which is known to mediate RAF-MEK1 binding for MEK1 phosphorilation (Therrien et al., 1996[52]). KSR1 protein levels were increased in IKKɛ- or TPL2-deficient DLD-1 cells (Figure 2A(Fig. 2), lanes 5-12). No such trend was observed in IKKɛ-deficient SW480 but not HCT116 cells (data not shown). We reasoned that the increased KSR1 levels may be related to decreased TPL2 kinase activity in IKKɛ deficient cells. However, there was no difference in TPL2 mRNA expression levels in SW480 or Wnt-transformed IECs (from *β-cat^c.a^*^. ^mice) lacking IKKɛ (Figure 2C&D(Fig. 2)). Therefore, we deduced that IKKɛ may interfere with TPL2 stabilization and its activity on downstream substrates via post-translational means. On the other hand, RAF was still another candidate for regulating MEK1 activation in tumor cells. To investigate this possibility, we performed Ras-activity assay in Wnt-transformed IEC samples lacking IKKɛ and we found that Ras/RAF1 activation was not affected (Figure 2E(Fig. 2)). Although these findings can account for residual MEK1 activation in the absence of IKKɛ, we can still speculate that the Ras/RAF axis may not be primarily involved in constitutive ERK1/2 phosphorylation in tumor cells. Therefore, IKKɛ may provide an alternative axis of MEK/ERK1/2 constitutive activation other than the very well-known signal-dependent Ras-RAF-MEK1 axis. To further explore the link between IKKɛ and ERK1/2, we also checked expression of ERK1/2-dependent MAPK phosphatases (MKPs), feedback inhibitors of MAPK signaling, from Wnt-transformed mice IECs (Figure 2F(Fig. 2)). To our surprise, many of these phosphatases were transcriptionally upregulated regardless of the absence of ERK1/2 phosphorylation in these samples (Supplementary Table 1). We also confirmed the upregulation of some of these phosphatases (SPRY1, SPRY2 and DUSP4) from the same samples via real time PCR (RT-PCR) experiments (Figure 2G(Fig. 2)). 

### TPL2 directly interacts with IKKɛ and this interaction requires IKKɛ kinase domain

**Figure 3:**
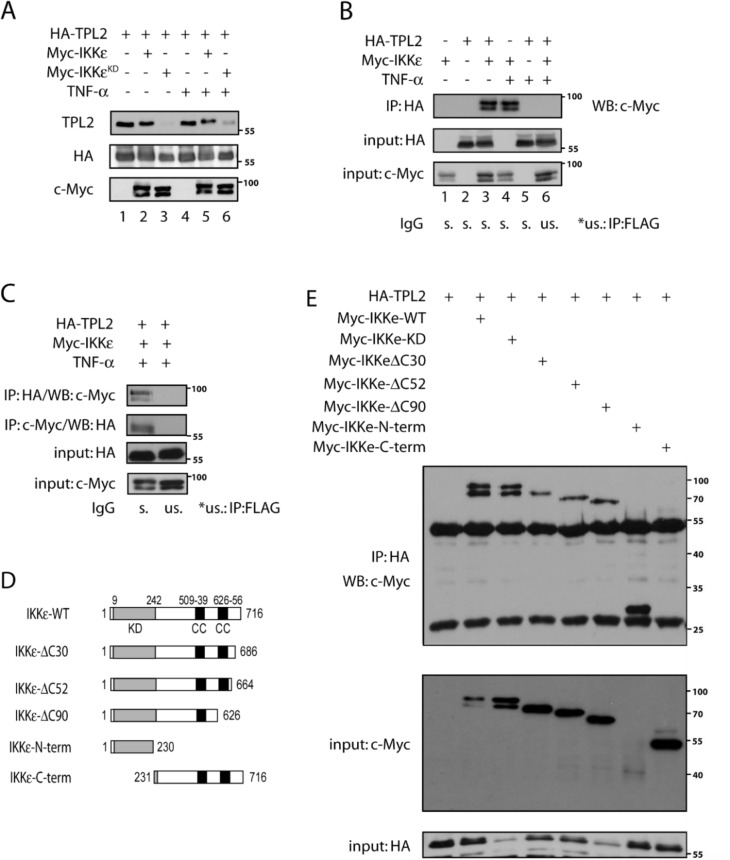
IKBKE interacts with MAP3K8 and their interaction is dependent on IKBKE kinase domain (N-terminal). (A) HA-TPL2, Myc-IKKɛ or Myc-IKKɛ-KD containing plasmids were ectopically expressed in HEK293 cells which are further stimulated 1 hour with 20 ng/ml TNF for IKKɛ/TPL2 activation. Then, protein samples were examined with WB by the help of indicated antibodies. (B) HEK293 cells were transfected with plasmids containing HA-TPL2 or Myc-IKKɛ constructs as indicated in the figure. Also, cells were stimulated 1 hour with 20 ng/ml TNF for IKKɛ/TPL2 activation. Then HA-TPL2 proteins were immunoprecipitated (IP) by the help of anti-HA antibody and co-immunoprecipitated IKKɛ is monitored by WB using the anti-Myc antibody. (C) With the same setting as before, a reciprocal IP experiment is carried using both anti-HA and anti-Myc precipitated proteins. (D) A sequence map showing different IKKɛ constructs which were used in further experiments for the identification of the IKKɛ domain required for the binding to TPL2. (E) HEK293 cells were transfected with different Myc-IKKɛ constructs (IKKɛ-WT, IKKɛ-KD, IKKɛ-ΔC30, IKKɛ-ΔC52, IKKɛ-ΔC90, IKKɛ-N-term, IKKɛ-C-term) and HA-TPL2, as indicated in the figure. Then, an IP experiment was carried on each sample using the anti-HA antibody and interacting IKBKE constructs were identified by WB.

Next, we explored whether the inverse correlation between IKKɛ and TPL2 protein levels are resulting from a direct interaction between both kinases. We could not see any interaction of endogenous proteins in tumor cells (data not shown). However, ectopically expressed IKKɛ and TPL2 associated in HEK293 cells (Figure 3A(Fig. 3)), independently of stimulation with 20 ng/ml TNF, an IKKɛ-activating cytokine. Interestingly, TPL2 was modified upon IKKɛ expression, possibly through phosphorylation. Reciprocal co-IP studies confirmed the association of both kinases (Figures 3B and 3C(Fig. 3)). To define the IKKɛ domain required for this interaction, we tested the capacity of a variety of IKKɛ mutants (IKKɛ-WT, IKKɛ-KD, IKKɛ-ΔC30, IKKɛ-ΔC52, IKKɛ-ΔC90, IKKɛ-N-term and IKKɛ-C-term) (Figure 3D(Fig. 3)) to bind TPL2. The IKKɛ-C-term mutant, which lacks the kinase domain, failed to bind IKKɛ, while all mutants, which still harbor the kinase domain, still bound TPL2 (Figure 3E(Fig. 3)). Therefore, the IKKɛ kinase domain is critical for the binding to TPL2 and this association may promote IKKɛ-dependent TPL2 phosphorylation.

### TPL2 and MEK1 are phosphorylated by IKKɛ 

**Figure 4:**
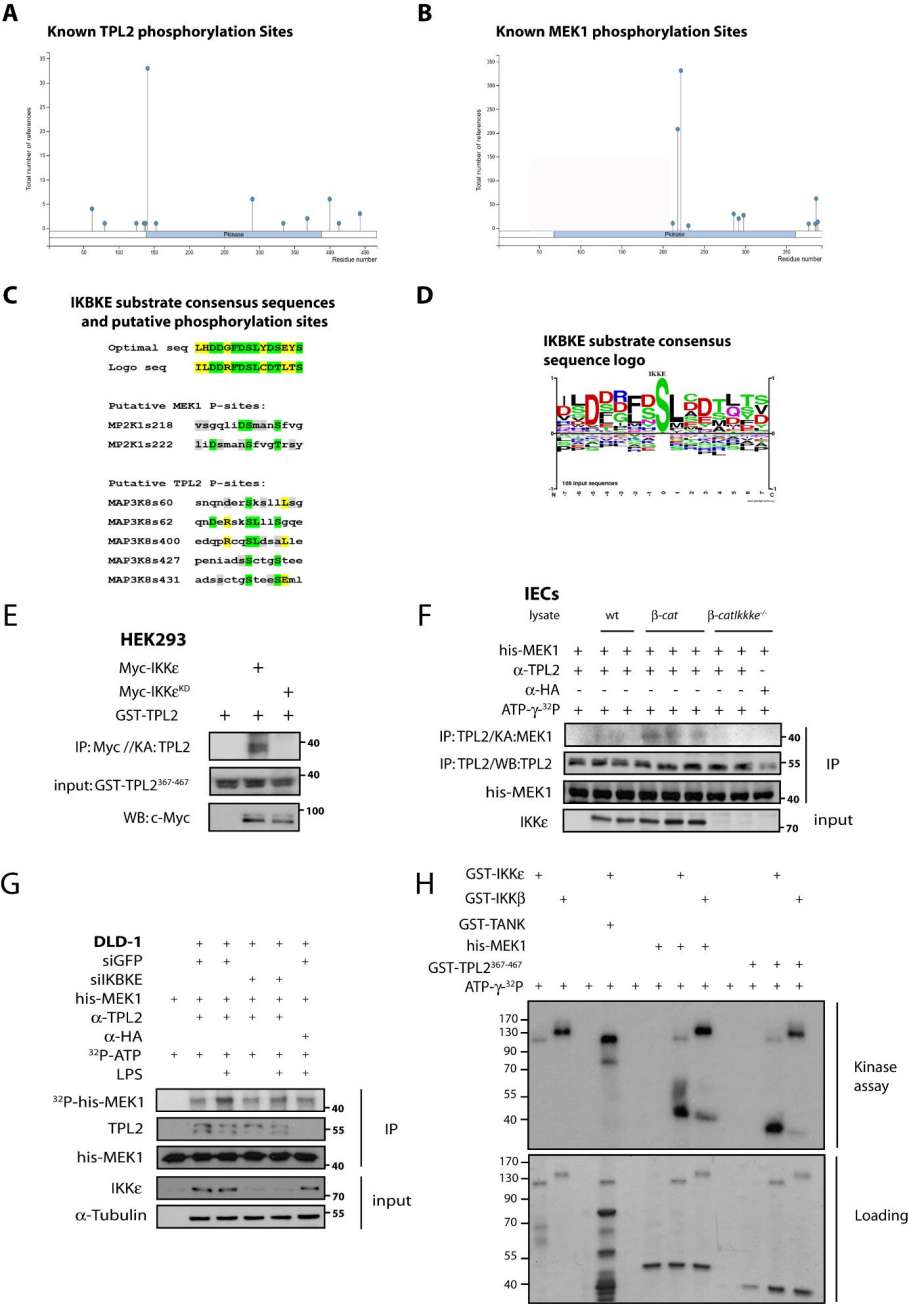
IKBKE phosphorylates TPL2 and MEK1 substrates *in vitro* and MEK1 phosphorylation by MAP3K8 depends on IKBKE. Known phospho-regulatory sites on (A) TPL2 and (B) MEK1. (C) Putative IKKɛ phosphorylation sites on TPL2 and MEK1 were determined by Phosphonet Kinase Predictor (Kinexus) and these sites were compared to optimal substrate consensus sequences for IKKɛ. (D) Optimal phosphorylation sites from known IKKɛ substrates analyzed and some potential phosphorylation sites were determined from Phosphosite algorithm and substrate consensus sequence logo is generated. (E) *In vitro* kinase assay has been performed with the overexpressed WT or kinase dead (KD) Myc-IKKɛ pulled out from HEK293 cells, using GST-TPL2^367-467^ as substrate. (F) Similarly, endogenous TPL2 is IPed from *β-cat^c.a^*^. ^vs *β-cat^c.a^*^.^-*Ikbke^-/-^* IEC lysates and used for an *in vitro* kinase assay against his-Mek1^KD^ substrate. (G) Another *in vitro* kinase assay was performed using si-scramble vs. siIKBKE transfected DLD-1 cells; endogenous TPL2 is immunoprecipitated and used for phosphorylation of his-Mek1^KD^ substrate. IKKɛ/TPL2 kinase activities in DLD-1 cells were stimulated via 0-1 hour 10 ng/ml LPS stimulation as indicated. (H) Recombinant GST-IKKβ and GST-IKKɛ were used for *in vitro* kinase assays to compare their kinase activity on GST-TANK, GST-TPL2^367-467^ and his-MEK1^KD^ substrates.

We next further explored whether TPL2 is phosphorylated by IKKɛ. First, we defined known phosphorylation sites on TPL2 and MEK1 (Figures 4A and 4B(Fig. 4)). Then, we analyzed the potential phosphorylation sites on IKKɛ substrates by the help of information on the known substrates (Figures 4C and 4D(Fig. 4)). Optimal phosphorylation site on substrates was determined by the help of the available information at Phosphosite Plus website (Hornbeck et al., 2015[23]). Then, we looked at potential IKKɛ phosphorylation sites suggested by Phosphonet (http://www.phosphonet.ca) Kinase Predictor (Kinexus, Canada) on both TPL2 and MEK1. More specifically, S68 on TPL2 and S218 on MEK1 and surrounding amino acid sequences showed an overlap with the optimal consensus sequence for IKKɛ while other possible sites (S400 on TPL2 and S222 on MEK1) also showed some similarity (Figure 4C(Fig. 4)). Therefore, both proteins were possibly targeted for phosphorylation by IKKɛ. To experimentally assess these possibilities, we performed *in vitro* kinase assay using extracts from HEK293 cells in which IKKɛ-WT or IKKɛ-KD proteins were ectopically expressed and immunoprecipitated. These IPs were used to assess the phosphorylation of a GST-TPL2^367-467 ^substrate. Only IKKɛ-WT but not IKKɛ-KD phosphorylated this substrate (Figure 4E(Fig. 4)). Next, endogenous TPL2 was also IP-ed from Wnt-transformed (*β-cat^c.a^*^. ^mice) IEC lysates (*Ikbke^wt^* vs. *Ikbke^ko^*) to perform an *in vitro* kinase assay using his-Mek1-KD as a substrate and where similar results were obtained in the absence of IKKɛ (Figure 4F(Fig. 4)). In a parallel experiment, lysates from DLD-1 cells transfected with siRNAs against scramble or IKKɛ and also stimulated with LPS were IP-ed with an anti-TPL2 antibody to collect endogenous TPL2 (Figure 4G(Fig. 4)). The immunoprecipitates were used in an *in vitro* kinase assay to assess the phosphorylation of the His-Mek1-KD substrate. We observed a marked decrease in TPL2 kinase activity in the absence of IKKɛ (Figure 4G(Fig. 4)). Therefore, both these experiments put forward that the ability of TPL2 to phosphorylate MEK1 is diminished without IKKɛ. Lastly, the kinase activity of recombinant GST-IKKɛ and GST-IKKβ were compared on GST-TANK, GST-TPL2 and his-MEK1-KD substrates in an in vitro kinase assay (Figure 4H(Fig. 4)). Although recombinant GST-IKKɛ phosphorylated a control substrate (GST-TANK) very weakly, IKKɛ phosphorylated GST-TPL2 and his-MEK1-KD very strongly and more specifically relative to GST-IKKβ. Since some MEK1 phosphorylation sites were potentially found to be phosphorylated by IKKɛ from the phosphosite in silico analyses, we also assessed whether IKKɛ could target MEK1 in this experiment. These observations collectively suggested that TPL2 kinase activity may be positively regulated by IKKɛ and that TPL2 and MEK1 can be specific substrates for IKKɛ for the maintenance of ERK1/2 constitutive activations in tumor cells.

**Figure 5:**
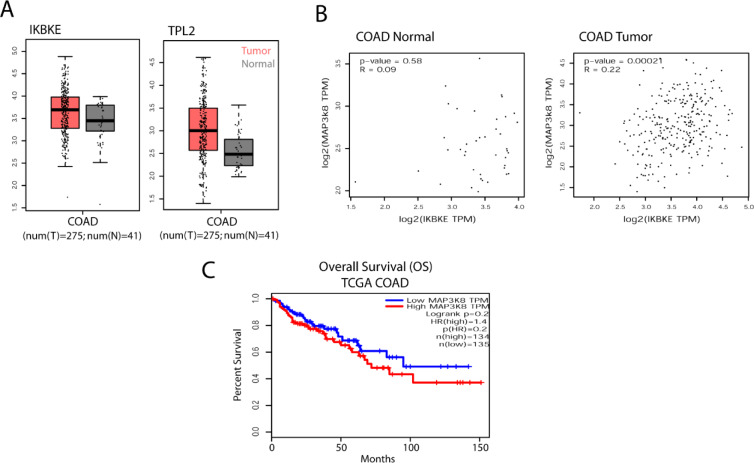
IKKɛ vs TPL2 expressions in TCGA colon adenocarcinoma (COAD) patient samples. (A) Tumor vs normal tissue IKKɛ and TPL2 expressions in TCGA COAD patient samples were presented in scatter plot and boxplot (no significant differences between tumor vs. normal tissue were observed). (B) IKKɛ/TPL2 co-expressions were analyzed by Pearson correlation coefficient in GEPIA where expressions of the genes were only significantly correlated in tumor samples. (C) COAD patient overall survival (OS) Kaplan-Meier graphs (KM-plot) were obtained for TPL2 high vs. low patients according to TCGA data in GEPIA (no significant difference was observed).

Finally, possible relation between IKKɛ and TPL2 expressions were also checked in TCGA patient samples for colon adenocarcinoma (COAD) (Figure 5(Fig. 5)). Although both genes were observed to be expressed more in tumor samples, these differences were not statistically significant (Figure 5A(Fig. 5)). Then, the co-expression of these genes was analyzed by the help of GEPIA tools (Tang et al., 2017[50]) in normal and tumor samples and only in tumor samples a significant correlation was observed (Pearson correlation coefficient (cc) R=0.22, p=0.00021) suggesting that co-expression of these genes is only relevant in tumor cells (Figure 5B(Fig. 5)). Finally, overall patient survival (OS) in TCGA COAD patients was analyzed in GEPIA via Kaplan-Meier survival plot (KM-plot) according to TPL2 expression (Figure 5C(Fig. 5)). Although a tendency of bad prognosis with high TPL2 expression was observed, the results were not yet reaching to statistical significance (Logrank p=0.2, HR=1.4). Therefore, we can conclude that the effect of IKKɛ on TPL2 and ERK1/2 activations are only due to post-translational modifications and not reflected to gene expression patterns in tumors. Finally, all these findings were summarized in a diagram related to IKKɛ-driven ERK1/2 activation in tumor cells (Figure S2). Accordingly, IKKɛ may reinforce ERK1/2 activation by direct phosphorylation of TPL2 and possibly by MEK1. Besides, IKKɛ may crosstalk with canonical Ras/Raf/MAPK signaling via yet to be identified mediators such as MKPs.

## Discussion

Most carcinomas are dependent on the activation of RTK/Ras/MAPK pathways for survival and proliferation. As a result, RTK signaling pathways are often dysregulated in tumor cells. There are many different ways of alterations these pathways that may cause similar outcomes in cellular responses (Lemmon and Schlessinger, 2010[33]). While dysregulations in canonical Ras/Raf/MAPK signaling is very common in many carcinomas, there are still other alternative paths for MEK/ERK activations in some tissues (Lavoie et al., 2020[28]). 

Numerous studies showed a TPL2-driven MEK/ERK1/2 activation pathway relevant in a number of cancers although the tumor-promoting or anti-tumor effect of TPL2 involvement is varying from one to another (Lee et al., 2015[30]). However, recent literature supports increasing number of tumor-promoting roles for TPL2 in different cancers addicted to RTK/Ras signaling. To illustrate, targetable TPL2 rearrangements were found in some melanoma tumor samples where no other driver mutations are known (Lehmann et al., 2019[32]). Moreover, TPL2 is a potential marker for predicting the outcome of MEK inhibitor treatment in high-grade ovarian cancers (Gruosso et al., 2015[20]). Similarly, some computational models suggested that TPL2 mediates Vemurafenib resistance in thyroid cancer cells (Gianì et al., 2019[15]). These last two examples are especially important since it supported our findings in IKKɛ/TPL2 creating a possible alternative route even in BRAF or MEK inhibitor-resistant tumors. Further support to these ideas has come from triple-negative breast cancer where IKKɛ was found to collaborate with MEK to drive TNBC development (House et al., 2018[24]). Similarly, IL-17 was found to induce cellular transformation in endothelial cells via TPL2 upregulation (Kim et al., 2013[26]). Given that IKKɛ regulates IL-17-dependent signaling in lymphocytes, adipocytes and intestinal epithelia (Bulek et al., 2011[3]; Göktuna et al., 2016[19]; Lee et al., 2017[31]), all these observations suggest that TPL2 can be the missing link for connecting IKKɛ to ERK1/2 activation in tumor cells. These observations do not rule out the possibility of IKKɛ to directly phosphorylate MEK1, as suggested by kinase assay with all recombinant proteins. Further experimental support is needed to strengthen this notion.

Alternatively, we also investigated the possibility of Ras-RAF involvement in IKKɛ deficient tumor cells. However, neither Ras-activity or KSR1 protein levels were found to be affected. Especially, KSR1 stabilization is important for mediating RAF-MEK binding for downstream ERK activation and KSR1 deficiency was found to prevent Ras-driven oncogenicity in mice (Lozano et al., 2003[37]; Therrien et al., 1996[52]). Moreover, KSR1 can also mediate MEK1 activation directly by phosphorylation (Goettel et al., 2011[16]) or MEK can allosterically regulate BRAF activation via KSR1 (Lavoie et al., 2018[29]). Given that TPL2 can antagonize KSR1 activity on MEK1 in leukemia cells (Wang and Studzinski, 2011[57]) and KSR2, a KSR1 related protein, can negatively regulate TPL2 activity in HeLa cells (Channavajhala et al., 2003[4]), there may be a competition or antagonism between both proteins for MEK1 occupancy or activation. Although TPL2 levels were found to be robustly increased in various CRC cell lines, this increase did not impact KSR1 protein levels unlike in leukemia cells. Therefore, regardless of the increased protein levels of both proteins, neither of them could deliver the activation of the MEK1 or ERK1/2 in the absence of IKKɛ. Collectively, these results support that the presence of IKKɛ is essential for ERK1/2 activation in tumor cells.

Given that Raf, Raf and KSR1 activities were not directly affected by the loss TPL2 kinase activity in IKKɛ-deficient tumor cells, there may be other mechanisms for the inhibition of canonical Ras/RAF/MAPK activation. To clarify this, we also investigated the expression of MAPK feedback inhibitors, namely MAPK phosphatases (MKPs). In addition to our observations with IKKɛ-dependent MEK1 and TPL2 activations, we also showed that some MKPs and some Sprouty phosphatases (SPRY1 and SPRY4) were also upregulated in CRC tumors deficient in IKBKE. Although most of these phosphatases act as feedback inhibitors for regulating RTK/MAPK signaling, their involvement can be different from one tissue to another (Bermudez et al., 2010[2]). While MKPs usually act as tumor suppressor due to their feedback inhibitory roles in RTK/MAPK signaling, they can be upregulated by various stress stimuli resulting in alternative MAPK activation such as JNK or p38 (Bermudez et al., 2010[2]). Given that IKKɛ deficiency abrogates AKT and ERK1/2 activations, transcriptional activation of Dusp family of MKPs in IKKɛ-deficient cells may be due to their essential roles in stimulating cell survival as previously shown for Dusp9 in squamous cell carcinoma (Liu et al., 2007[36]). On the other hand, Sprouty phosphatases were described as regulators or Ras and RAF activities (Hanafusa et al., 2002[22]; Sasaki et al., 2003[45]). However, later research on these phosphatases showed that upregulation of Spry1 or Spry2 is essential for providing a positive feedback loop on ERK1/2 activity and silencing of these proteins resulted in tumor regression (Schaaf et al., 2010[46]) including in BRAF-mutated melanomas (Montico et al., 2020[40]).

Eventually, the effect of IKKɛ control on MEK/ERK1/2 activation does not only depend on IKKɛ specific substrates (like TPL2 and MEK1) but also depends on IKKɛ-regulated expression of the phosphatases (both of which may depend on IKKɛ kinase activity). These dysregulations of phosphatase expressions may be due to some IKKɛ-dependent inhibition on transcription factors which in turn control the transcription of these phosphatase genes. Therefore, the impact of IKKɛ loss in a cell is dual: impaired MEK1 and ERK1/2 phosphorylations and dysregulated transcription of MAPK feedback inhibitors, both of which render tumor cells unable to activate ERK1/2/MAPK pathway. Eventually, this may also suggest that there may be other possible cross-talk mechanisms between IKBKE and Ras/RAF signaling for the regulation of ERK1/2 and downstream targets in a tissue or cell specific manner. Nevertheless, to elucidate the nature of such interactions further and more comprehensive studies are required. 

If IKKɛ provides an alternative mechanism to reinforce MEK/ERK1/2 activation in tumor cells, then the suspending question is what can be the physiological meaning for this alternative pathway. While RAS/RAF-driven ERK1/2 activation is essential in signal-dependent ERK1/2/MAPK activation in many different tissues, the constitutive RAS or ERK1/2 activation may lead to senescence or apoptosis in wild type but not in mutated p53 cells (Ferbeyre et al., 2002[12]; Lin et al., 1998[34]; Lin and Lowe. 2001[35]; Serrano et al., 1997[47]; Tang et al., 2002[49]). Therefore, tumor cells may need to find alternative ways to operate ERK1/2 activation to further proliferation and survival without activating senescence or apoptosis (Deschênes-Simard et al., 2013[8]). One solution to this problem can be the activation of alternative MEK/ERK1/2 upstream kinases like TPL2. Hence, IKBKE-driven TPL2/MEK1 activation may present an opportunity to further ERK1/2 activations without triggering p53-driven apoptosis in tumor cells. To illustrate this issue, KSR1 ablation reverted oncogenic Ras-mediated senescence in MEF cells (Kortum et al., 2006[27]). Given that a possible antagonism between TPL2 and KSR1, as we aforementioned, TPL2-mediated alternative ERK1/2 activation may evade p53 and p16(ARF)-mediated senescence for tumor cell to continue proliferation. Similarly, a recent study in pancreatic ductal adenocarcinoma (PDAC) has put forward that TPL2 enforced the activation of RAS-induced inflammatory signals to ensure constitutive ERK1/2 phosphorylation for tumor cell survival (Dodhiawala et al., 2020[10]). In this study, they also suggested that targeting TPL2 may overcome resistance against RAF or MEK inhibitors in pancreatic cancer as well. As described in our previous studies (Göktuna et al., 2016[19]), IKKɛ may play an essential role in tumor cell survival in early tumorigenesis. Regardless of the absence of AKT and ERK1/2 activations in IKKɛ-deficient cells, there was no impact on proliferation of colorectal cancer cells. Therefore, we can speculate that IKKɛ-driven ERK1/2 activation is primarily required for the survival of Wnt-transformed cells in the genotoxic tumor microenvironment. Although apoptosis was avoided in tumor cells due to their ability to trigger NF-κB-dependent anti-apoptotic machinery in the presence of IKKɛ, it is also possible that IKKɛ -driven MAPK activation may help tumor cells to avert p53-driven apoptosis or senescence. However, this question is beyond the scope of this study and further experimental support is still needed to clarify this possibility. 

In summary, all these findings strongly support our hypothesis that IKKɛ-driven and TPL2-dependent alternative ERK1/2-activating path may be mainly required for cell survival in tumor cells. Eventually, IKKɛ-driven TPL2 and MEK1 phosphorylations and MKP transcriptional regression support a feedforward loop to maintain an alternative route of constitutive ERK1/2 activation which may be crucial for cell proliferation, survival and chemotherapy resistance in tumors. 

## Declaration

### Conflict of interest

The author declares that he has no conflict of interest with the contents of this article.

### Author contributions

S. I. G. conceptualization; S. I. G. investigation; S. I. G. funding acquisition; S. I. G. formal analysis; S. I. G. writing original-draft.

### Acknowledgments

I thank Prof. Alain Chariot (University of Liège) for all the support and resources he has provided; Prof. Florian Greten (GSH, Frankfurt University, Germany) for providing *Vil-Cre-ER^T2^-Ctnnb1^+/lox(ex3)^* mice, Dr. Emmanuel Dejardin (GIGA, University of Liège, Belgium) for providing MEF-T cells, Mrs. Servin Bagheralmoosavi for technical help, Dr. Onur Çizmecioğlu (Bilkent University, Turkey) for valuable discussions and Dr. Tieu Lan Chau (Bilkent University, Turkey) for critical reading of the manuscript.

### Funding and additional information

Studies reported here were supported by F.N.R.S, FLF and WELBIO funds in Belgium and TÜBİTAK ARDEB 116Z349 grant in Turkey.

### Data availability statement 

RNA-Seq data from a previous publication was used for re-analysis (Göktuna et al., 2016[19]). All this information is contained in the referred article and the related data is available upon request.

### Supplementary information 

This article includes supplementary information.

## Supplementary Material

Supplementary information
