# Supplementary material for: IKBKE-driven TPL2 and MEK1 phosphorylations sustain constitutive ERK1/2 activation in tumor cells
Source: EXCLI J. 2022 Feb 18;21:436–53. doi: 10.17179/excli2021-4578 (PMC8983855; doi:10.17179/excli2021-4578)
Supplement: Supplementary information [file EXCLI-21-436-s-001.pdf]

*EXCLI Journal 2022;21:436-453 – ISSN 1611-2156*
*Received: December 07, 2021, accepted: February 17, 2022, published: February 18, 2022*

# Original article:

# IKBKE-DRIVEN TPL2 AND MEK1 PHOSPHORYLATIONS SUSTAIN CONSTITUTIVE ERK1/2 ACTIVATION IN TUMOR CELLS

Serkan İsmail Göktuna[1,2,3]

[1] Department of Molecular Biology and Genetics, Bilkent University, Ankara, Turkey
[2] National Nanotechnology Research Center (UNAM), Bilkent University, Ankara, Turkey
[3] Laboratory of Medical Chemistry, Interdisciplinary Genomics and Genoproteomics Research Center (GIGA), University of Liege, Liege, Belgium

* **Correspondence to:** Serkan İsmail Göktuna, Department of Molecular Biology and Genetics, Bilkent University, 06800 Bilkent, Ankara, Turkey.
E-mail: serkan.goktuna@bilkent.edu.tr

https://dx.doi.org/10.17179/excli2021-4578

This is an Open Access article distributed under the terms of the Creative Commons Attribution License (http://creativecommons.org/licenses/by/4.0/).

**Supplementary Table 1: MKP expression data related to Figure 3.** RTK/MAPK phosphatases whose expression is significantly regulated among 2179 differentially expressed transcripts in Wnt-driven transformed IECs (data related to Figure 2G).

| Gene | Fold Change ($Ikbke^{ko}/Ikbke^{wt}$) | $log_2$(Fold Change) | Rank (in 2179 transcripts) |
|---|---|---|---|
| **Spry4** | 4.79 | 2.26 | 147 |
| **Dusp9** | 3.14 | 1.65 | 293 |
| **Dusp14** | 2.48 | 1.6 | 401 |
| **Dusp3** | 1.93 | 0.94 | 638 |
| **Dusp7** | 1.92 | 0.94 | 639 |
| **Spry1** | 1.70 | 0.74 | 800 |
| **Dusp4** | 1.52 | 0.61 | 944 |
| **Dusp5** | 0.70 | -0.51 | 1522 |

EXCLI Journal 2022;21:436-453 – ISSN 1611-2156
Received: December 07, 2021, accepted: February 17, 2022, published: February 18, 2022

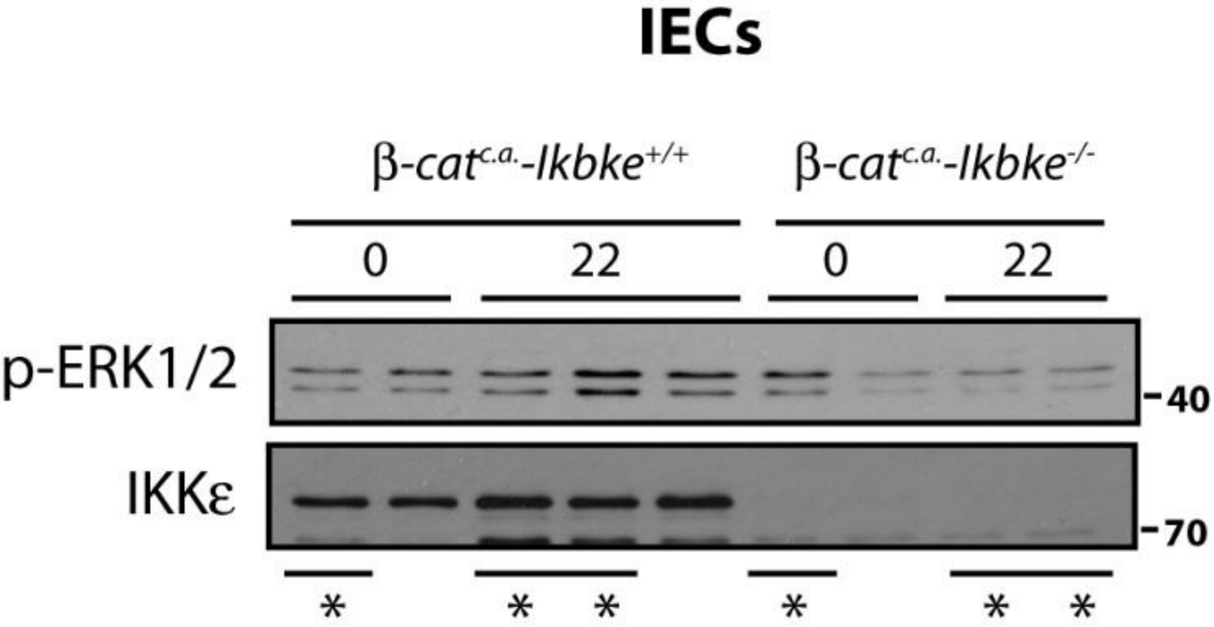


**Supplementary Figure 1: Supplementary unedited WB scans related to Figure 2.** Unedited WB images showing loading of the included samples for the active-Ras assay (*samples included in active-Ras assay in Figure 2E).

EXCLI Journal 2022;21:436-453 – ISSN 1611-2156
Received: December 07, 2021, accepted: February 17, 2022, published: February 18, 2022

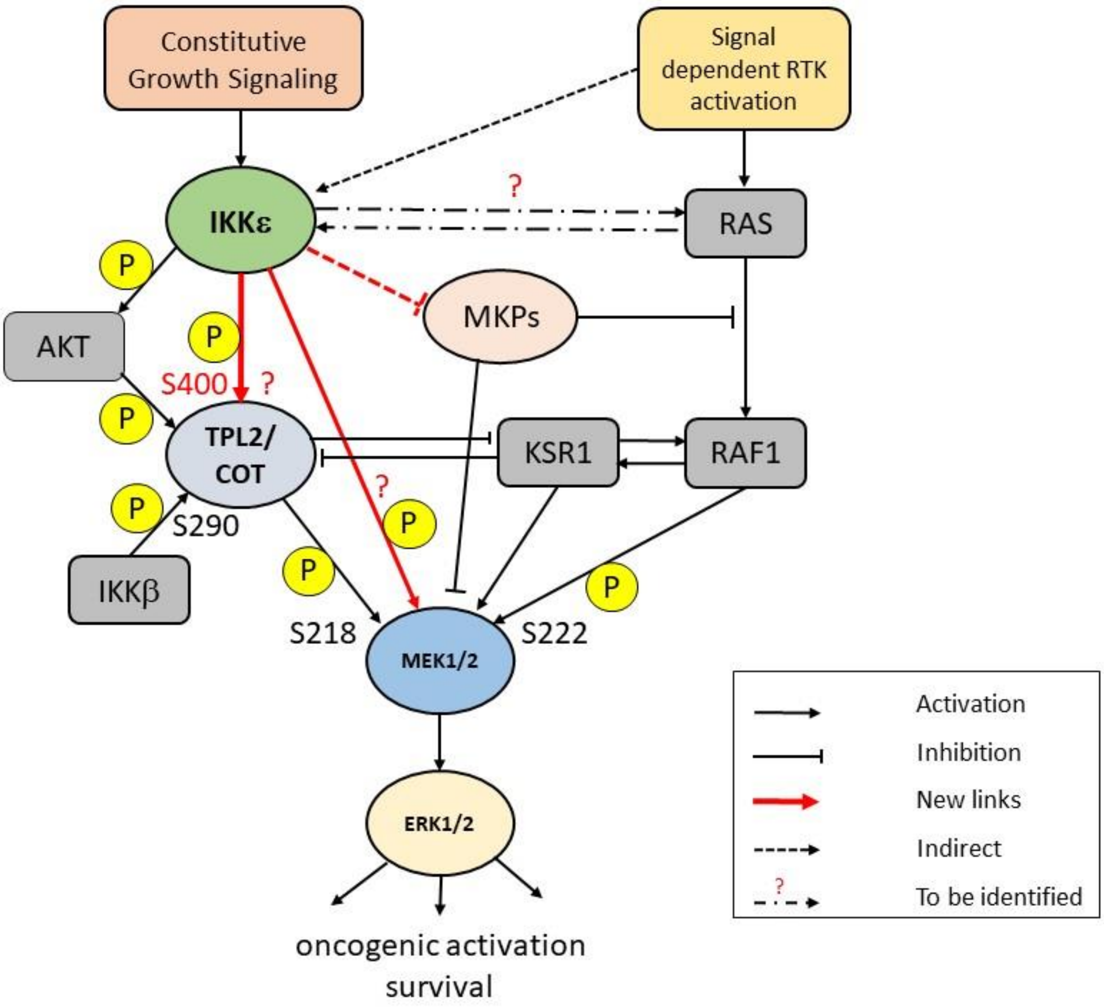


**Supplementary Figure 2: IKKε regulated ERK1/2 activating signaling networks.** IKKε reinforce ERK1/2 activation in transformed cell via direct phosphorylation of TPL2 and MEK1. IKKε driven AKT and IKKβ-driven TPL2 activations may also help to ensure constitutive MEK and ERK1/2 activation. Alternatively, oncogenic Ras-driven RAF and MEK activations may also account for constitutive ERK1/2 activation in transformed cells. IKKε may help to maintain this pathway via indirectly inhibiting MKP upstream activators. However, IKKε -driven TPL2 activation may antagonize KSR1 in transformed cells most probably to provide an alternative survival path to avoid oncogene activated senescence and cell death.

*Received: December 07, 2021, accepted: February 17, 2022, published: February 18, 2022*

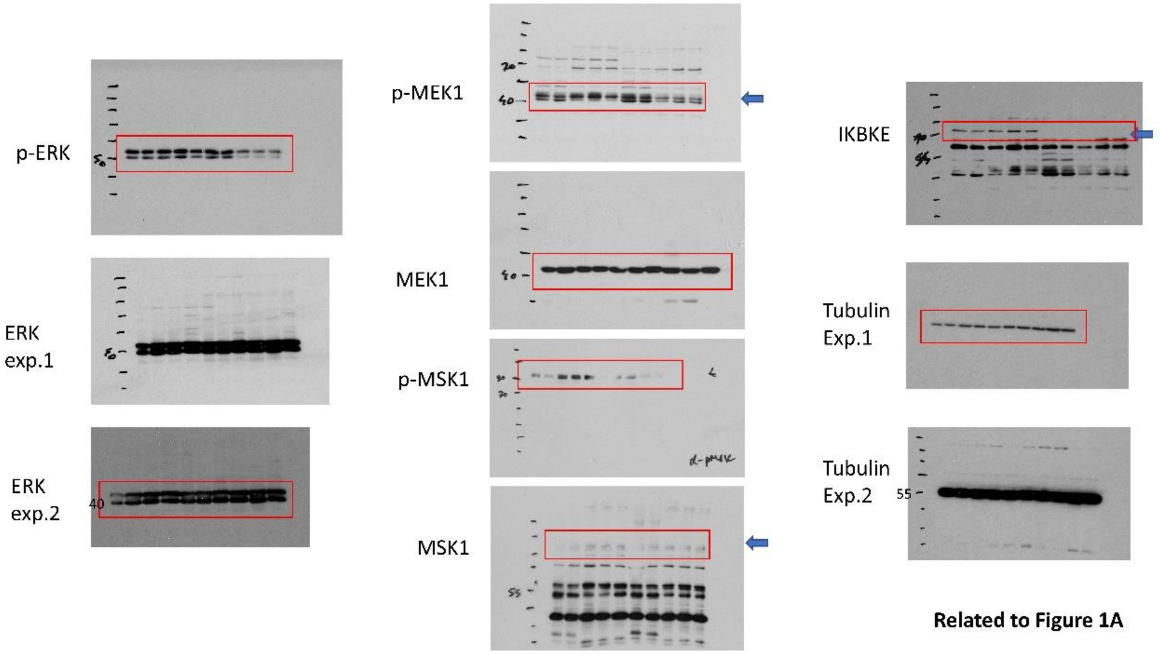


**Supplementary Figure 3: Whole WB scans related to Figure 1A. "**Exp.1" and "Exp. 2"; different exposures for the same WB. Only indicated exposures with the red boxes were used in the main figures.

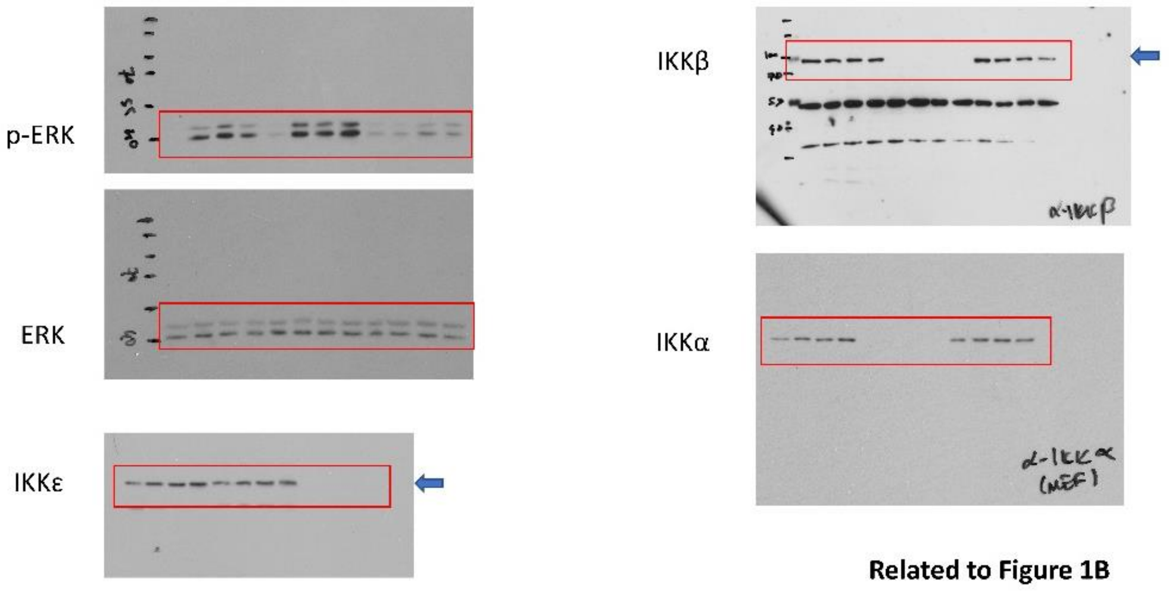


**Supplementary Figure 4: Whole WB scans related to Figure 1B.** Only indicated exposures with the red boxes were used in the main figures.

*EXCLI Journal 2022;21:436-453 – ISSN 1611-2156*
*Received: December 07, 2021, accepted: February 17, 2022, published: February 18, 2022*

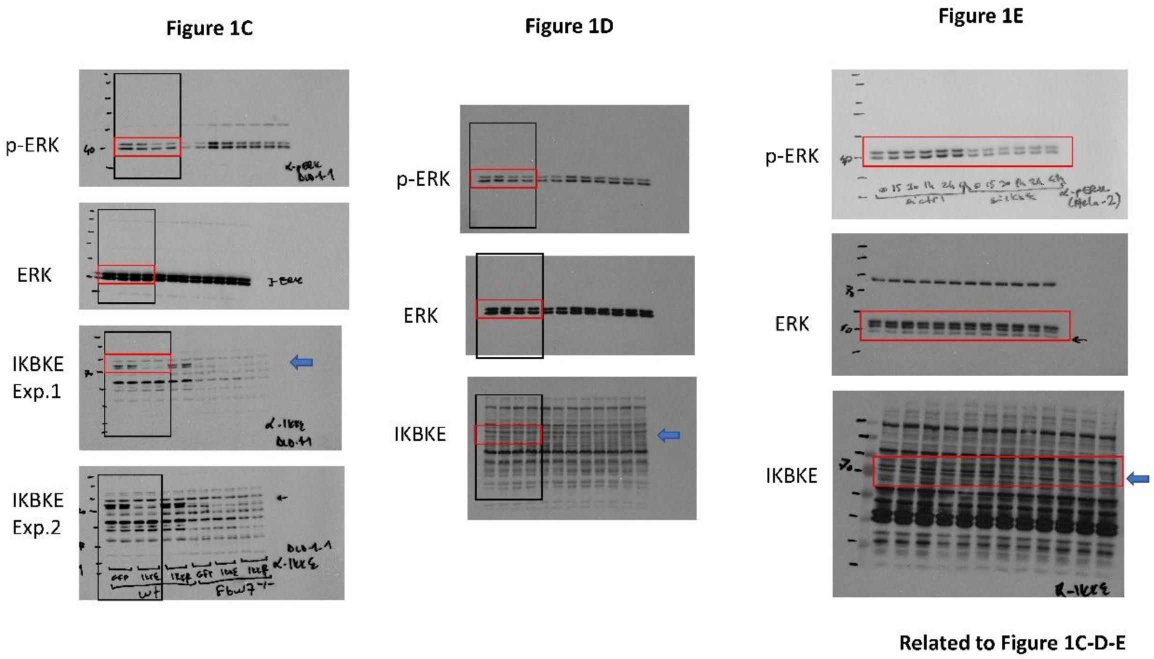


**Supplementary Figure 5: Whole WB scans related to Figure 1C-E. "**Exp.1" and "Exp. 2"; different exposures for the same WB. Only indicated exposures with the red boxes were used in the main figures. Black boxes indicate relevant samples for the given experiments (where some additional samples from an unrelated experiment may be loaded as well).

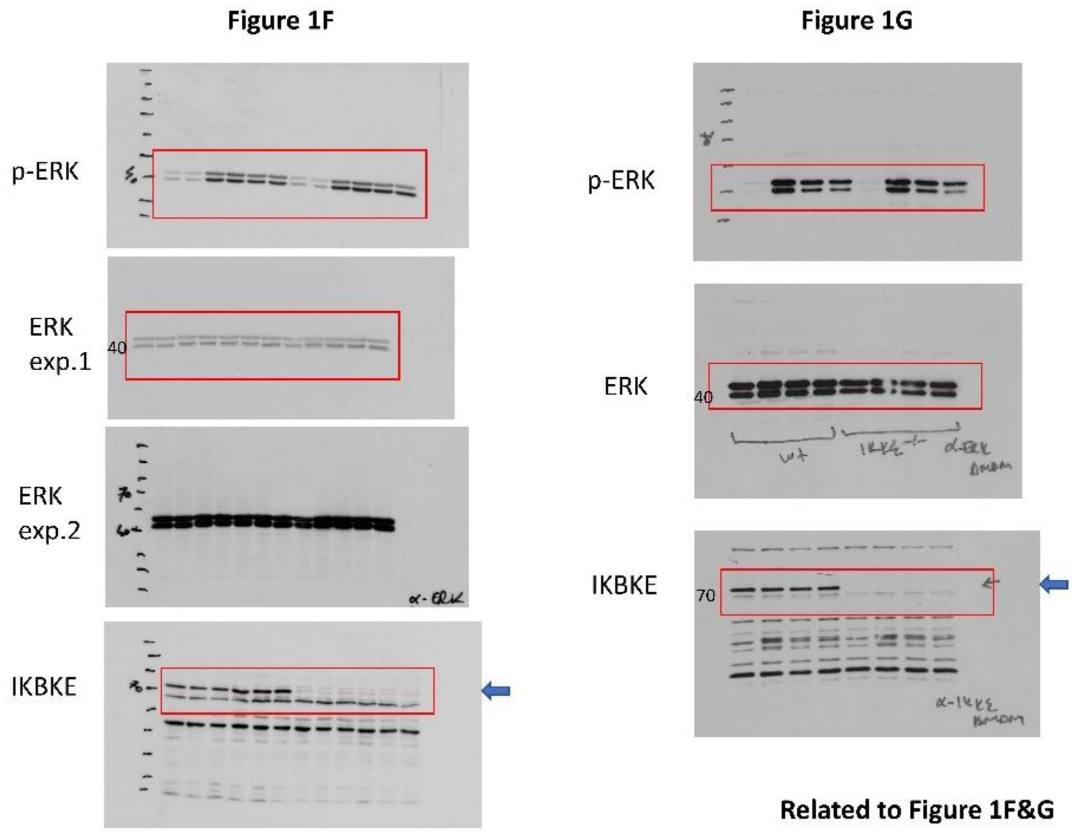


**Supplementary Figure 6: Whole WB scans related to Figure 1F&G. "**Exp.1" and "Exp. 2"; different exposures for the same WB. Only indicated exposures with the red boxes were used in the main figures.

*EXCLI Journal 2022;21:436-453 – ISSN 1611-2156*
*Received: December 07, 2021, accepted: February 17, 2022, published: February 18, 2022*

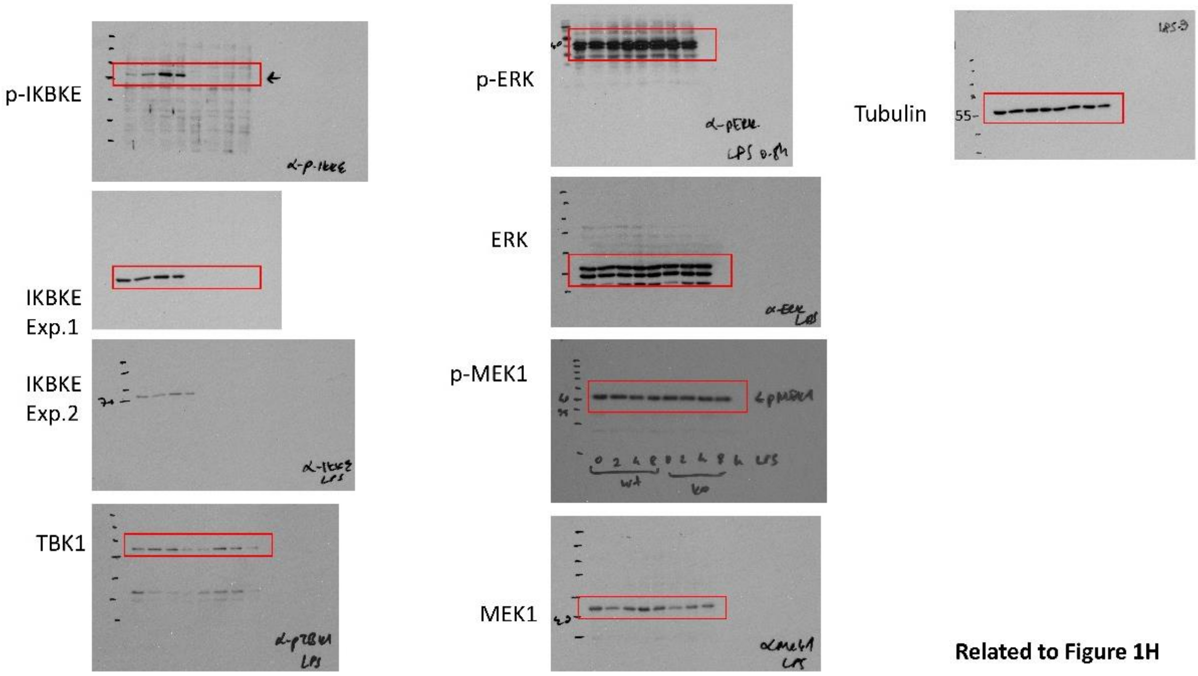


**Supplementary Figure 7: Whole WB scans related to Figure 1H. “**Exp.1” and “Exp. 2”; different exposures for the same WB. Only indicated exposures with the red boxes were used in the main figures.

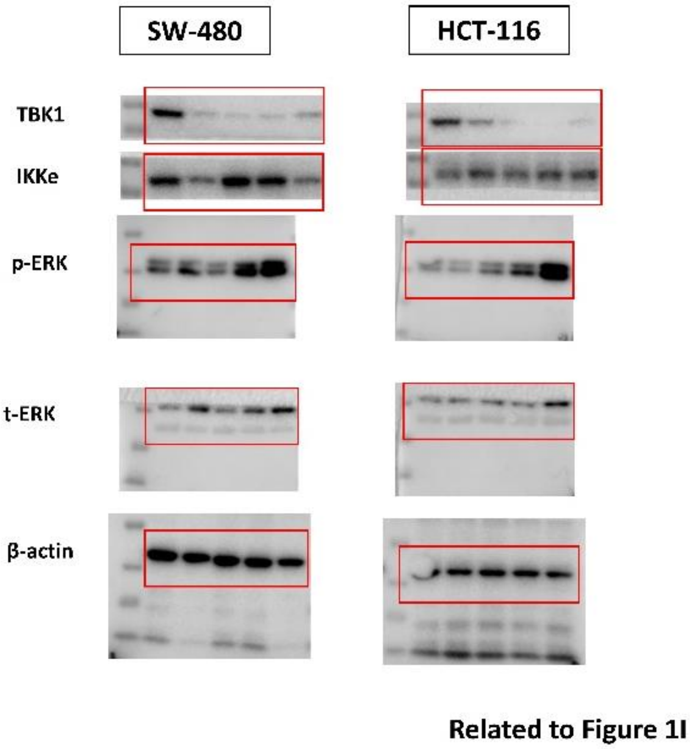


**Supplementary Figure 8: Whole WB scans related to Figure 1I.** Only indicated exposures with the red boxes were used in the main figures.

EXCLI Journal 2022;21:436-453 – ISSN 1611-2156
Received: December 07, 2021, accepted: February 17, 2022, published: February 18, 2022

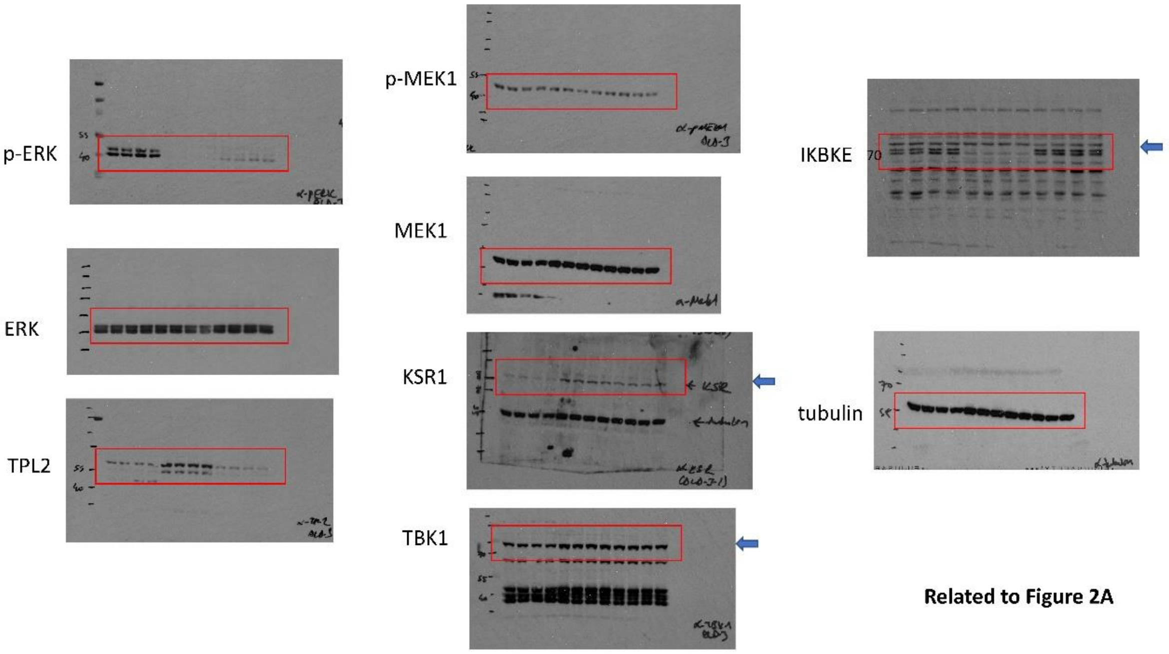


**Supplementary Figure 9: Whole WB scans related to Figure 2A.** Only indicated exposures with the red boxes were used in the main figures.

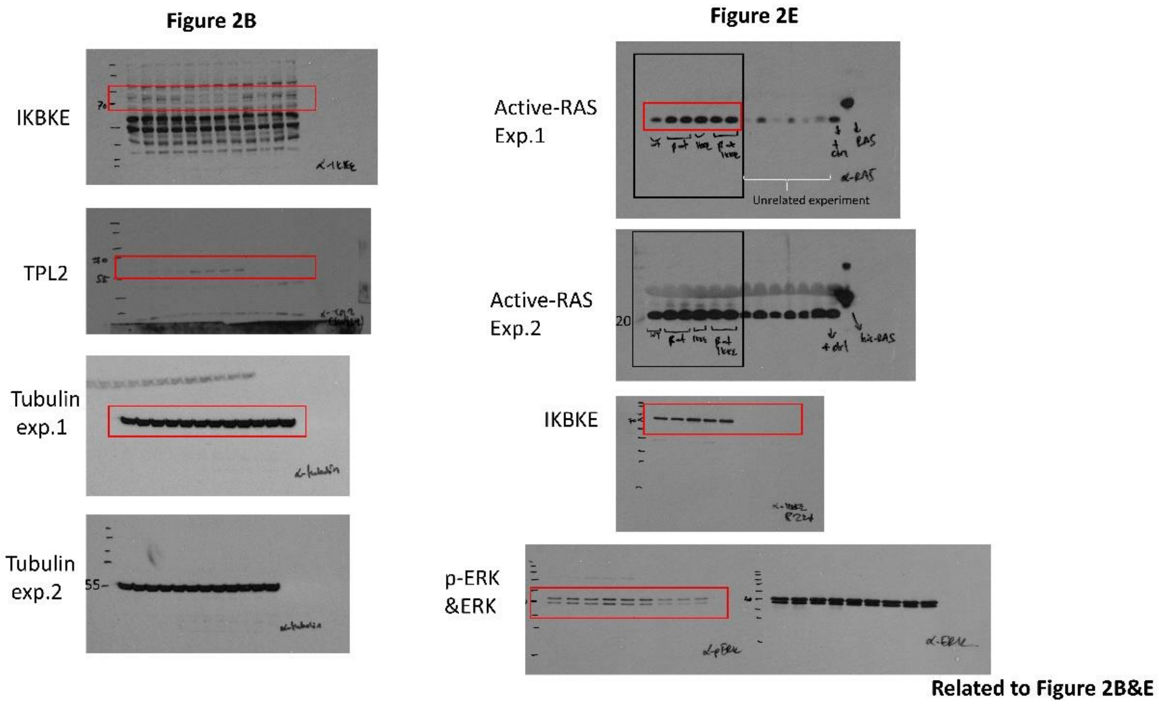


**Supplementary Figure 10: Whole WB scans related to Figure 2B&E. "**Exp.1" and "Exp. 2"; different exposures for the same WB. Only indicated exposures with the red boxes were used in the main figures. Black boxes indicate relevant samples for the given experiments (where some additional samples from an unrelated experiment may be loaded as well).

*EXCLI Journal 2022;21:436-453 – ISSN 1611-2156*
*Received: December 07, 2021, accepted: February 17, 2022, published: February 18, 2022*

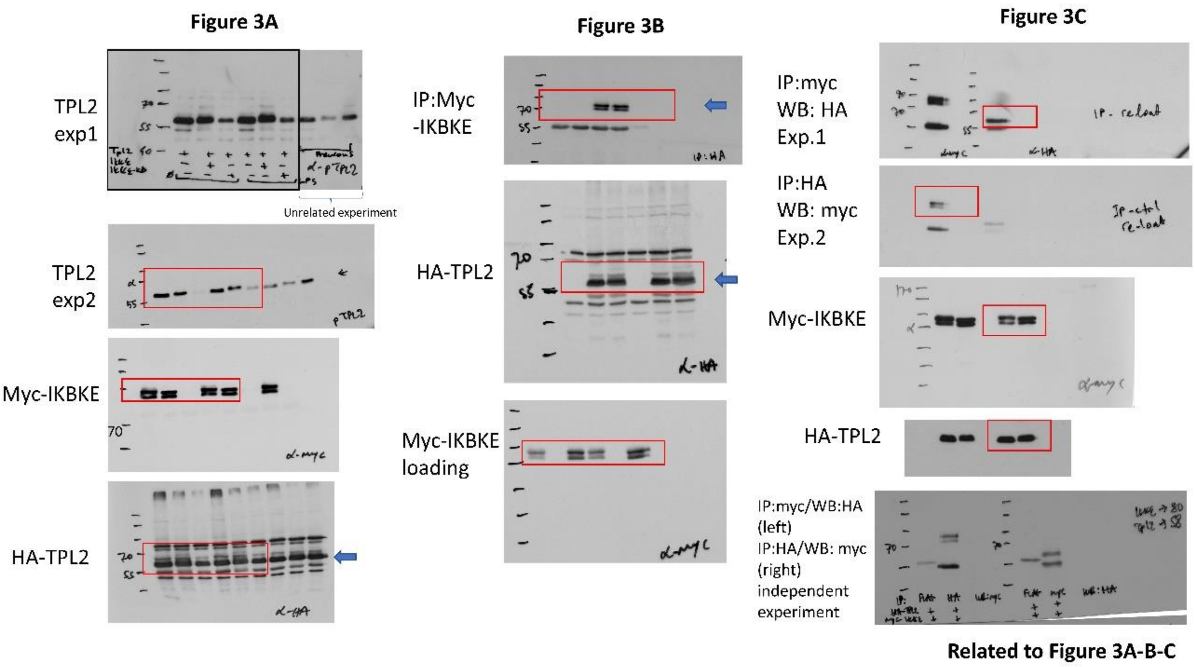


**Supplementary Figure 11: Whole WB scans related to Figure 3A-C. “**Exp.1” and “Exp. 2”; different exposures for the same WB. Only indicated exposures with the red boxes were used in the main figures. Black boxes indicate relevant samples for the given experiments (where some additional samples from an unrelated experiment may be loaded as well).

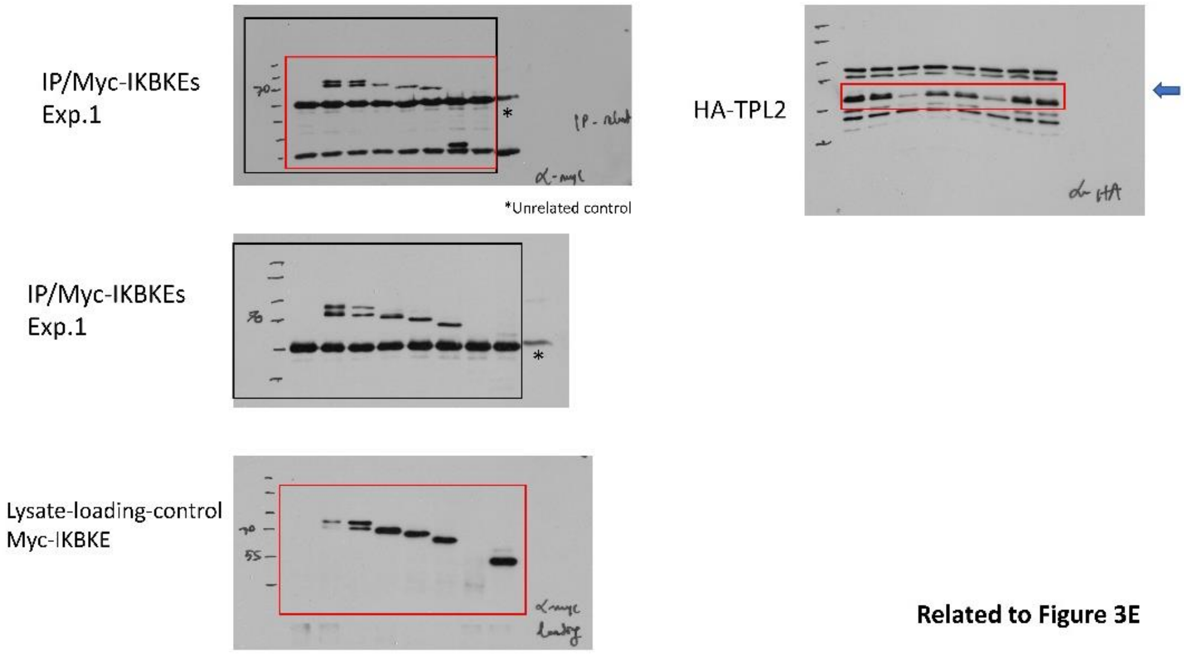


**Supplementary Figure 12: Whole WB scans related to Figure 3E. “**Exp.1” and “Exp. 2”; different exposures for the same WB. Only indicated exposures with the red boxes were used in the main figures. Black boxes indicate relevant samples for the given experiments (where some additional samples from an unrelated experiment may be loaded as well).

*EXCLI Journal 2022;21:436-453 – ISSN 1611-2156*
*Received: December 07, 2021, accepted: February 17, 2022, published: February 18, 2022*

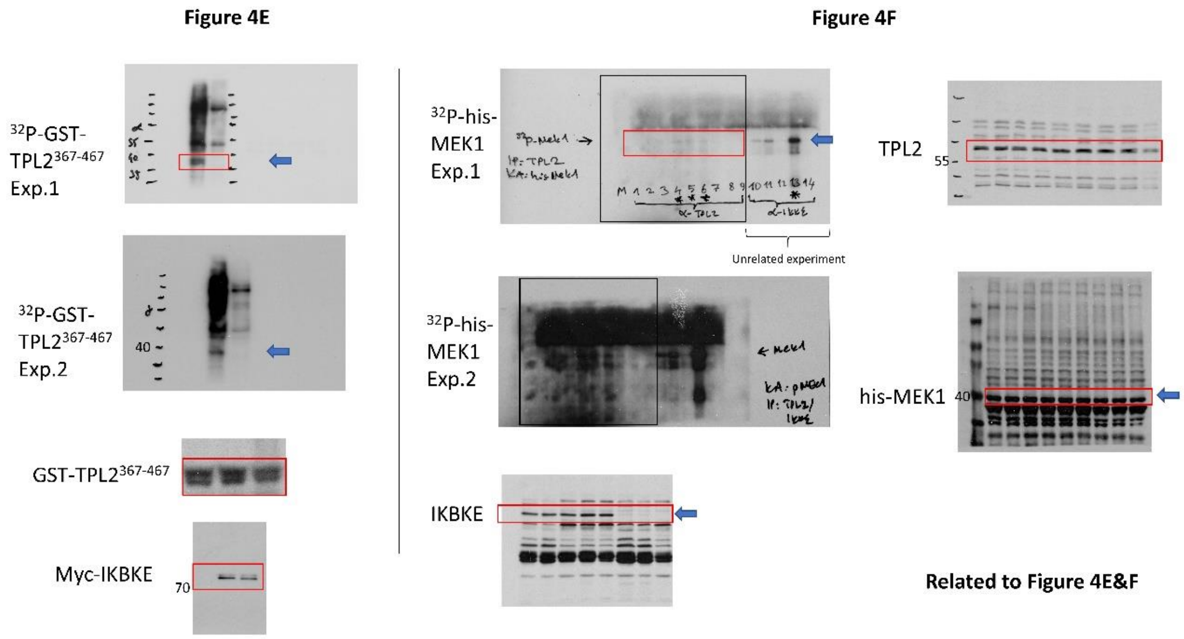


**Supplementary Figure 13: Whole WB scans related to Figure 4E&F.** “Exp.1” and “Exp. 2”; different exposures for the same WB. Only indicated exposures with the red boxes were used in the main figures. Black boxes indicate relevant samples for the given experiments (where some additional samples from an unrelated experiment may be loaded as well).

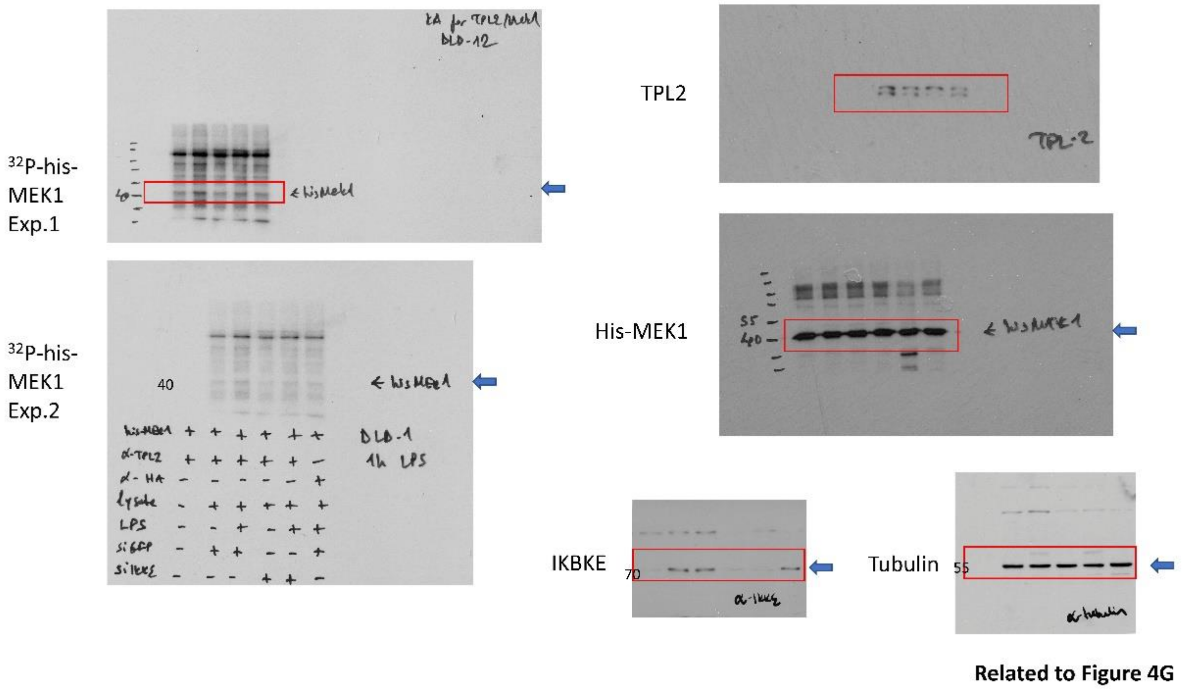


**Supplementary Figure 14: Whole WB scans related to Figure 4G.** Only indicated exposures with the red boxes were used in the main figures.

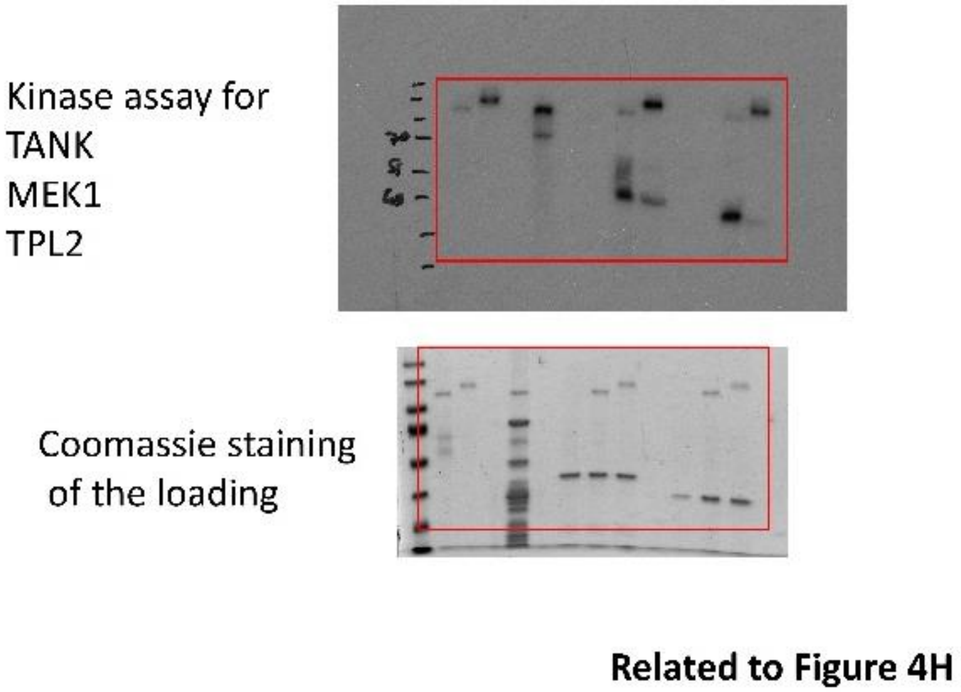


**Supplementary Figure 15: Whole WB scans related to Figure 4H.** Only indicated exposures with the red boxes were used in the main figures.
